# Peer support for accepting distressing reality: Expertise and experience-sharing in psychiatric peer-to-peer group discussions

**DOI:** 10.1177/13634593231156822

**Published:** 2023-02-27

**Authors:** Elina Weiste, Melisa Stevanovic, Lise-Lotte Uusitalo, Hanna Toiviainen

**Affiliations:** Finnish Institute of Occupational Health, Finland; Tampere University, Finland; University of Helsinki, Finland; Tampere University, Finland

**Keywords:** conversation analysis, epistemics, expert-by-experience, peer support, psychiatry

## Abstract

Peer-based interventions are increasingly used for delivering mental health services to help people with an illness re-examine their situation and accept their illness as part of their life story. The role of the peer supporter in these interventions, known as experts-by-experience (EbE), is situated between mutual peer support and semi-professional service delivery, and they face the challenge of balancing an asymmetric, professional relationship with a reciprocal, mutuality-based, equal relationship. This article investigates how EbEs tackle this challenge when responding to clients’ stories about their personal, distressing experiences in peer-based groups in psychiatric services. The results show how the EbEs responded to their clients’ experience-sharing with two types of turns of talk. In the first response type, the EbEs highlighted reciprocal experience-sharing, nudging the clients toward accepting their illness. This invoked mutual affiliation and more problem-talk from the clients. In the second response type, the EbEs compromised reciprocal experience-sharing and advised clients on how to accept their illness in their everyday lives. This was considered less affiliative in relation to the client’s problem description, and the sequence was brought to a close. Both response types involved epistemic asymmetries that needed to be managed in the interaction. Based on our analysis, semi-professional, experience-based expertise involves constant epistemic tensions, as the participants struggle to retain the mutual orientation toward peer-based experience-sharing and affiliation.

## Introduction

Serious mental illness constitutes a biographical disruption in a person’s life story. The illness can have long-standing consequences for the structures of a person’s everyday life and the forms of knowledge that underpin them (Bury, 1982). The illness affects the person’s family and wider social relations, disrupting the normal rules of reciprocity and mutual support. A person with an illness has to learn to increasingly depend on others. Further, they are forced to tolerate the uncertainty of their situation and to re-examine their own expectations and plans for the future (Bury, 1982).

Receiving social support from people who have faced, endured, and overcome the same adversity in life has shown to evoke a sense of hope, affecting positive changes in various life domains (e.g. Davidson et al., 2012). This is especially the case when the peer support involves positive self-disclosure, role-modeling, and conditional regard (Davidson et al., 2012). The idea is that a peer may help the person re-examine their situation and accept the illness as a part of their life story by sharing their own first-hand experiential knowledge of illnesses and their treatment in service systems. This appreciation of experiential expertise has given rise to a new group of people who are trained as “peer support workers” or “experts-by-experience” (EbEs) to work alongside professionals in service encounters and service development processes (Jones, 2021).

EbEs are individuals with a lived illness who have constructed a story of the personal experiences, actions, and issues that have helped them on their journey, typically through specific training (Jones and Pietilä, 2020). This personal growth process from a “patient” to an “expert” may help these people re-contextualize their past experiences and construct professionalized identities (Jones and Pietilä, 2020). Often this process requires learning to tell your story in an “analytical way” which involves taking emotional distance from it, but, at the same time, describing your emotions in a way that makes them recognizable and identifiable for others to relate to (Toikko, 2016). This type of generalized experience, coming from the individuals’ own experiences, may then be collectively shared and create mutual understanding, affiliation, and empowerment (Mead et al., 2001). This kind of reciprocity has been suggested as unattainable in client–professional relationships (Munn-Giddings and Borkman, 2017).

In mental health services, peer support delivered by EbEs can be conceptualized as “involving one or more persons who have a history of mental illness and who have experienced significant improvements in their psychiatric condition offering services and/or supports to other people with serious mental illness who are considered to be not as far along in their own recovery process” (Davidson et al., 2006: 444). Unlike mutual support programs, peer-based interventions are thus closer to the mainstream of mental health practice, involving an asymmetrical relationship with at least one designated support provider and one designated support recipient (Davidson et al., 2006). Thus, EbEs face a challenge: they need to balance an asymmetric, professional type of relationship with a reciprocal, mutuality-based equal relationship. Our interest in this article lies in unraveling how EbEs tackled this challenge during and through social interaction in a peer-based group intervention.

From the perspective of social interaction, expertise-by-experience is essentially about claiming and displaying *epistemic authority* or *epistemic rights* (Heritage and Raymond, 2005; Stevanovic and Svennevig, 2015) in a certain field of specialist knowledge (Mikkonen and Saarinen, 2018: 42). Clients have direct, first-hand knowledge of their experiences of illness and its treatment, which cannot be replaced by medical professionals’ superior general knowledge of medical symptoms and their causes. At the same time, this very field of specialist knowledge allows EbEs to become equally positioned with the clients, as they are expected to share the same experiences of the illness. The notion of equality has been argued as holding, to the extent that the EbE–client relationship can also become healing and empowering to the peer supporter, not only to the peer recipient (Clay, 2005). Thus, while attempts to even out epistemic asymmetries are an essential aspect of many encounters between clients and professionals (Halonen, 2008; Weiste et al., 2016), we may assume that EbEs have less need to do so.

As has been demonstrated in conversation analytic (CA) studies on *epistemics* (Heritage, 2011), implicit claims and displays of knowledge are an omnirelevant feature of talk. Although they may become particularly relevant in some contexts, such as those of advice-giving (Peng, 2022; Vehvilainen, 2001), and expert assessment (Lee, 2018), they are also part of talk that is at first glance not at all related to claiming and displaying knowledge. Experience-sharing is a prime example of the latter (Enfield, 2011; Stevanovic and Frick, 2014). Heritage (2011) investigated everyday storytelling and showed that experience-sharing is in effect a truly dilemmatic endeavor, as participants’ orientations to their own and each other’s epistemic rights tend to impose constraints on their expressions of empathy and affiliation, which are nonetheless essential for successful experience-sharing. Whereas the lack of a substantial response to a story is inherently problematic for the teller, the provision of such a response may result in the recipient assuming a competitive position in relation to that of the story-teller.

The dilemmatic aspects of experience-sharing may become even more acute during interactions between EbEs and clients. In such encounters, the experiences shared by the clients typically involve depictions of problems, which make advice-giving highly relevant as a response. Whereas in a multitude of other institutional settings, the provision of advice would be the expected way of responding to clients (e.g. Weiste et al., 2021), here, this is not the case. Instead, EbEs are instructed to refrain from advice-giving and instead to respond to the client’s telling from the perspective of their own analogous experiences as peers (Hietala, 2016). Thus, in addition to the above-mentioned generic dilemmas associated with all conversational experience-sharing, this experiential epistemic asymmetry may lead to another type of problem. It may guide the participants’ interpretations of each other’s conduct away from experience-sharing and toward the advice-giving frame in which EbEs are more knowledgeable than clients.

In this study, we examined sequences of interaction in which clients tell EbEs about their experiences and the EbEs respond to the clients. Our investigation targets two different loci in these sequences. We ask:

RQ1: How do EbEs treat and respond to clients’ experiential telling when working toward acceptance of the clients’ difficult life situations?
RQ2: How do clients react to the ways in which the EbEs receive their tellings?

In our investigation of these two questions, we paid particular attention to the subtle acts of balancing an asymmetric relationship and expressions of expertise and an equal relationship and reciprocal experience-sharing.

## Data and method

This study was based on a dataset of three audio-recorded, peer-based group discussions (total of 3.5 hours of interaction) collected as a part of the Social and health care professionals as experts on client involvement project. The discussions were held on 1 day in two hospital wards (HW1 and HW2) and one outpatient unit (OPU) of a municipal health care district in Finland. The discussions were led by the EbEs, and no professionals were present. The hospital had a long tradition of using EbEs for developing organizational practices and organizing peer support for clients. In the units, the peer support sessions took place once a week in a day room. Participation in the sessions was voluntary and the number of clients varied significantly across the sessions (HW1 one client, HW2 two clients, OPU seven clients). The clients treated in the units were aged between 18 and 65 and suffered from severe psychiatric conditions. The sessions were led by the same working pair of EbEs, who were experienced in guiding peer support sessions and had undergone formal training as EbEs by the hospital district (see Jones, 2021 for more about the training).

The general aim of the peer support sessions was to provide clients with a safe, confidential environment in which to talk. There were no predefined topics, but the sessions were advertised as covering, for example, discussions on everyday coping skills, resources, hobbies, and weekly schedules. The sessions were different to treatment discussions in that no decision on treatment or medication was made during them. The EbEs had no access to patient records and did not write notes. Professionals were only informed about the discussions at the client’s wish. Each peer support session lasted approximately 1 hour. It started with the EbE introducing the idea of the session and emphasizing the confidentiality of the discussion. The discussion began by the EbE asking the clients how things were going or how their week had been. Next, the EbEs asked questions and talked about their own experiences, to invite the clients to talk. Most of the conversations were between the EbEs and a particular client. The session ended when the time was due, without any formal closing discussion.

Permission to collect data was obtained from the hospital district and Ethics Committee of Finnish Institute of Occupational Health (23 November 2018). The fourth author was present in the peer support discussions. She informed the staff of the research before the recordings and all the participants at the beginning of each session. Informed, written consent was obtained from all the participants, and they were advised that they could withdraw their consent at any point during the research process. When the EbEs opened the session, the researcher sat apart from the group without intervening in the discussions. The anonymity of the participants has been ensured by altering any details that may enable their identification.

The data were analyzed using CA which core idea is to examine recordings of naturally occurring interactions and unravel the recurring practices through which the social actions are accomplished. According to the CA view, social actions are organized into sequences of initiating and responsive actions (Schegloff, 2007). CA explores the relations between these sequential patterns, their turn design, and the subsequent development of talk (Drew et al., 2001). In this study, CA offered an apt methodology for identifying observable patterns of interaction which may facilitate reciprocal participation (see Drew et al., 2001) in peer-to-peer intervention. In practice, we listened to the recordings several times and identified all the sequences of talk in which a client told the EbE about their personal experiences and the EbE responded to this telling (29 instances). These instances were transcribed using CA conventions (Jefferson, 2004; see Appendix). The instances were quite evenly distributed between group discussions: nine were found from HW1 (two EbEs with one client), 12 from HW2 (two EbEs with two clients) and eight from OPU (two EbEs with seven clients). We analyzed all these instances case by case, paying specific attention to their design, the surrounding interactional context, and the ways in which the EbEs responded to the clients. We also analyzed the clients’ uptakes of the EbEs’ responses. By this way, we were able to show how the selections which EbEs made in designing their turns had certain consequences for how the interaction proceeded (see Drew et al., 2001).

## Results

The clients in our data shared their personal experiences via extensive turns during which they described the ambivalence and difficulty in their current life situations. The EbEs received the clients’ narrations and responded in two ways. In the first, the EbEs placed the clients’ experience into the general stages of the rehabilitation process and shared their own past experiences, which were often even more problematic than those that the clients had expressed. By sharing their solutions to these past problems, the EbEs invited the clients to talk more about their problems and to adopt accepting perspectives of their difficult situations. In the second response type, the EbEs explicitly advised the clients on how they should think and act to adopt more lenient thinking patterns about themselves. After such advising turns, the clients did not continue their narrations and the sequence was brought to a close.

In the following two subsections, we describe these two patterns in more detail, focusing first on two cases in which the clients and the EbEs orient toward mutually sharing distressing experiences. In the first case, the client complies with the EbE’s invitation and continues to describe her problems. In the second case, the client also continues to talk about the problem, even if subtle misalignments emerge. In the second subsection, we present a case in which the mutual experience-sharing is compromised, as the EbEs more unilaterally advise the client on how to think and act.

### Nudging toward acceptance of illness: Highlighting reciprocal experience-sharing

In response to the clients’ experiential telling, the EbEs shared their own difficult past experiences, showing affiliation with the clients’ situations. The experiences were placed into a more general frame of the rehabilitation process, involving a long temporal continuum from the past to the present, and showing the EbEs’ own initial difficulties in accepting the consequences of their illness. In talking about accepting the need for help and tolerating the lengthiness of the rehabilitation process, the EbEs invited the clients to talk more about their problems and to adopt more accepting, lenient attitudes toward their illness. The EbEs also talked about their own solutions to the mutually shared problems—either explicitly or implicitly, using idiomatic expressions.

In Extract 1, the client takes an ambiguous stance toward her experience and the EbE shares her analogous but temporally divergent and more distressing experience. Just before the extract, the client has told the EbE about her weekend visit to her parents’ house. She said that her young children were staying at her parents’ house while she was in hospital. During the visit, the client took care of her children and found it very enjoyable but also tiring.

**Extract 1** (HW2, two EbEs, two clients)

**Table:** 

01	CLI1:	ni se oli kyllä ihanaa vaikka
		*so it was really wonderful even though*
02		se oli tosi kuormittavaa ja mä olin
		*it was also really straining and I was*
03		sen jälkeen todella väsyny,
		*really tired afterwards*,
04	EbE1:	k y ll ä.
		*yes.*
05	CLI1:	ja k o ko ↑sen p äi vän olin ihan
		*and the whole day I was*
06		hirveen väsyny mut #kyllä siitä sai
		*really tired but #it definitely gave me more*
07		enemmänku mitä se sitten niin ku otti#.
		*more than it took out of me#.*
(removed 46 rows of conversation about fatigue)		
08	EbE2:	sitte siinä tulee kuitenki se että
		*but the fact is that*
09		kyllähän se kuntoutuminen (.)
		*yes the rehabilitation does (.)*
10		meillä jatkuu niin kun (.) tosi pitkään.
		*continue for us like (.) for really long.*
11		kellä se on joku määr ä tty? aika ja,
		*for some it’s a definite amount of time and*,
12		kellä se jatkuu tavalla tai toisella
		*for others it continues in one way or another*
13		loppuelämän, mutta se että (.)
		*for the rest of their lives, but the fact that (.)*
14		et sit siellä tulee niin kun niitä huonompia
		*there will be, like, some of the worse*
15		aikoja ja. #niin kun parempia aikoja
		*times and. #like better times*
16		ja muita ni# kyllä se sit niin
		*and so on# yeah it will be, like*,
17		kun lasten kanssa on (.) °on semmonen et°
		*with the kids (.)* °*it’ll be like*°
18		et kyl mulle on ollu sillo, (.) iso (.)
		*it was back then, (.) a major (.)*
19		iso tekijä niin ku se et mulla oli
		*major factor, like when*
20		sit siinä se (.) mies ja sitte meiän äiti
		*my (.) partner was there and my mother*
21		jotka sitte sillo autto. #autto# et nythän
		*who helped me then. #helped# and now*
22		nuo on jo niin isoja että sitte.
		*they (the kids) are so grown up that.*
23	EbE1:	niin se on sitte ku lapset kasvaa
		*yeah that’s what it’s like when kids grow up*
24		mutta tota. (.) °kyl siinä tietyssä
		*but well. (.) °at a certain*
25		vaiheessa ne vaatii kuitenki sen.°
		*phase they do demand it.°*
26	EbE2:	ja jotenki mun oli ainaki mun oli
		*and somehow it was - at least for me it was*
27		äärettömän vaikee hyväksyä sitä #omaa
		*exceptionally hard to accept my #own*
28		uupumusta ja sitä väsymystä#
		*exhaustion and the fatigue#*
29	EbE1:	joo siihen meni se oma aikasa ennen
		*yeah and it took time for*
30		ku sen niin ku.
		*me to, like.*
31	CLI1:	iteki vielä just mieltää itteään todella
		*I also still think of myself really*
32		helposti huonoks äidiks ku ei jaksa
		*easily as a bad mother when I don’t have the strength*
33		perusjuttuja ees tehä ni,
		*to do even the basic stuff so*,
34		että seki miten se sunnuntai,
		*also the way things went on that Sunday*,
		(removed 6 lines description on sunday happenings)
42		kyllä mun oli sanottava sitte siinä (.)
		*so I did have to say then (.)*
43		päivällisaikaa että mä en jaksa tehä
		*at dinnertime that I’m too tired*
44		tota ruokaa että voitteko te tehä tän.
		*to cook so could you do this.*
45	EbE1:	ja se on semmosta sitte jo tervettä itsekkyyttä.
		*and that’s sort of healthy selfishness.*
46	EbE2:	joo se on ↑viisautta.=
		*yeah it’s* ↑*wisdom.=*
47	EbE1:	=viisautta (.) myöntää että tota noin ni,
		=wisdom (.)admitting that it, well,
48	CLI1:	°alko olee niin uupunut° et kaikki
		*°I began to feel so exhausted° that everything*
49		rupes itkettää ni oli semmonen että nyt (.)
		*made me cry so it was like, now (.)*
50		#mä meen sohvalle#
		*#I’m going to lie down on the couch#*
51	EbE1:	et nyt on yliväsymys (.) kyllä.
		*that now you’re overtired (.) yeah.*
52		se on ihan totta (.) ja sit siinä
		*that’s so true (.) and then*
53		ainaki mulle niin ku helposti siinä
		*for me at least it easily*
54		tulee semmonen että vähän niin ku
		*becomes like a kind of must?*
55		se on se pakkoki? mikä sitten taas vie
		*which again wears out*
56		sitä (.)niitä voimavaroja kun niin ku
		*that (.) those resources when you*
57		yrittää yli voimavarojen. (.)
		*overdo things. (.)*
58		v(hh)ähän semmone (.) ↑oravanpyörä,
		*a bit (hh) like (.) ↑a rat race*,
59		et mullahan käy edelleenkin kotiapu (.)
		*I’m still getting domestic help (.)*
(removed 12 lines talk on EbE’s difficulties in receiving help)		
72		kunnes jossain vaiheessa se käänty niin päin
		*until at some point it turned into*
73		että se on mun #oikeus#
		*being my #right#*
74	CLI1:	°helpottaa°
		*°it gets easier°*
75	EbE1:	nii,
		*yeah*,

In the first lines, the client evaluates her day as wonderful (line 1) but also straining (line 2). She emphasizes being extremely tired (lines 3 and 6) and concludes that the visit “gave her more than it took out of her” (lines 6–7). By describing and evaluating her experiences, she presents herself as a person with direct, first-hand knowledge of the experiences that she is entitled to communicate (Heritage, 2011). After an extensive talk about the client’s tiredness and fatigue (not shown in the extract), EbE2 responds to their experience-sharing (line 8). She first re-frames the client’s telling by connecting it to the rehabilitation process and its longevity, which are characterized by fluctuations of better and worse periods (lines 9–15). By connecting the client’s experience to the phase of the rehabilitation process, EbE2 positions herself as someone who has knowledge of the process and can place the client’s particular experience into the larger frame. What is notable, however, is that EbE2 refers to those whose rehabilitation is a longstanding process as “us,” including herself as one of the rehabilitees (line 10). In this way, the epistemic relations become more even, as EbE2 can refer to her own experience as well as those of the client.

Next, in line 18, EbE2 explicitly shifts to talking about her own experience. She states that like the client, she received help with childcare, which was “a major factor” for her (lines 18–21). She emphasizes that—in contrast to the client’s situation—her experience is in the past. She not only talks in the past tense, but also refers to the past moment in time (“back then,” line 18) and highlights that her children are now grown-ups (line 21–22). EbE2 also states that accepting her exhaustion and fatigue (similar experiences to those the client talks about) were exceptionally difficult for her (lines 26–28). With the word *ainakin* (“at least,” line 26) she implies that this is how she felt, but that others might feel the same. Her experience is supported by EbE1, who claims to have had the same experience in the past (lines 29–31). Thus, both EbEs display access to the client’s experience on the basis of their own past experiences (Heritage, 2011). By describing their own problematic experiences, they show affiliation and invite the client to continue her telling.

Indeed, the client continues talking about her experience, adopting a problematic stance toward her weekend visit. She states that she felt like a “bad mother” as she had no strength to “do the basic stuff” (line 31–33). The client frames her experience as similar to that of the EbEs by using the clitic particle *-kin* (*itsekin*, “me too,” line 31). She provides a particular example of “being a bad mother” by describing how she became so tired after playing with her children that she needed to go and rest on the couch and ask her parents to make dinner (lines 42–44). At this point, both EbEs explicitly validate the client’s behavior, describing it as “healthy selfishness” (line 45) and “wisdom” (line 46). Moreover, EbE1 reformulates the client’s experience as “overtiredness,” which makes the client’s behavior not only understandable, but also legitimate (line 51), and even “true” (line 52). This type of formulation has been noted as typical in therapy interaction when the therapist displays access to the client’s experience (Weiste et al., 2016).

After validating the client’s experience, EbE1 immediately continues by telling the client about her own current problematic situation. She refers to her own experience with words *ainakin mulle* (“at least for me,” line 53) and describes how getting rest should be a must (line 53–55) when she exceeds her resources by overdoing things (56–57). She describes this as “a rat race” (line 58). Next, in line 59, she reveals that she is still receiving domestic help, relating her situation to that of the client. Her stance toward the help has changed, however. After describing her shame when she applied for domestic help every year (not shown in the extract), she is now able to consider such help as her “right” (lines 72–73). Thus, she claims to have finally accepted her fatigue and need for help. Showing how EbE1 has resolved the same challenging experience that the client has described in her current reality imbues the experience-sharing with epistemic asymmetry. The client nonetheless seems to take EbE1’s experience as comforting as she states quietly *helpottaa* (“it gets easier,” line 74), supposedly implying that, as the situation has become easier for EbE1, it will also become easier for her.

In sum, the EbEs shared their own distressing experiences of accepting their own need for help, and placed these troubles in a longer temporal continuum from the past to the present moment. In this way, they invited the clients to talk about their problems from more accepting, lenient perspectives. The EbEs also shared their solutions to their mutually shared problems, imbuing epistemic asymmetry. The client aligned with the EbEs’ actions and also shared their troubling thoughts.

In Extract 2, the client takes an ambiguous stance toward her situation, which the EbE then addresses from a more problematic perspective. In this case, however, the client misaligns with the EbE’s perspective shift and downgrades the relevancy of the problem in her case. Still, the participants retain the mutual orientation toward experience-sharing and affiliation.

Prior to the extract, the client has just shared her skills for coping when she feels distressed. Here she topicalizes smoking as a way of releasing anxiety and calm herself down (line 1).

**Extract 2** (HW2, two EbEs, two clients)

**Table:** 

01	CLI1:	sit ↓valitettavasti tupakanpoltto?
		*then* ↓*unfortunately smoking cigarettes?*
02		auttaa erittäin paljon,
		*is very helpful*,
03		°se on semmonen rauhottava (.)
		*It’s, like, calming (.)*
04		ei ehkä terveellin en,°=
		*maybe not healthy,°=*
05	EbE1:	=↑no e:i, [↓mut sen aika sitte]
		=↑well no:, [↓but its time will]
06	EbE2:	[no ei terveellinen niin]
		*[well not so healthy yeah]*
07	EbE1:	voi olla jossain myöhemmässä vaiheessa
		*may be at some later point*
08		miettiä että (.)voihan sitä
		*to think that (.) you can*
09		ajatusta lopettamisesta haudutella mutta (.)
		*let the thought of quitting brew but (.)*
10		ne asiat useesti menee vähän semmoseen?
		*those things usually go in a kind of?*
11		tärkeysjärjestykseen siinä vaiheessa?
		*order of importance at that stage?*
12	EbE2:	kyl mä ite ainaki muistan sillon ku oli
		*I do remember myself that when I was*
13		osas↑tohoidossa, ja (.)
		*in the hospital, and (.)*
14		tavallaan oli muutenkin ne huonoimmat
		*in a way those were also the worst*
15		(.) ajat (.) ehkä sen mielen
		*(.) times (.) perhaps with that mind*
16		<hallinnan> ja sen (.) mielen heittelehtimisen suhteen
		<*control*> *and (.) mood swings*
17		(1.8) >siis poltin itsekin sillon<
		(*1.8*) >*I* mean I smoked back then<
18		sillä sai sit niin ku sitte rauhotettua
		*it was a way of calming myself down*
19		ja vähän sitä ahdistusta pois joskus
		*and getting rid of that anxiety a bit sometimes*
20		jopa sillee kotona ku olin ↑et heräs yöllä
		*even when I was at home I woke up in the middle of the night*
21		#semmosee ahdistukseen ni sit
		*#to that anxiety and*
22		piti käydä yötupakalla# °tai°,
		*I had to go for a night smoke# °or°*
23	CLI1:	joo kyl mä niinku (.) mullekin tuli
		*yeah I, like (.) I also*
24		sen osastojakson jälkee
		*after the hospital period*
25		mä aloin aloin polttaa vasta- (1.0)
		*I started to smoke only- (1.0)*
26		vasta niin ku sit ku °jouduin jouduin
		*only when I ° was hospitalized in*
27		psykiatriselle osastolle° (2.0)
		*the psychiatric ward° (2.0)*
28		eli en oo en oo polttanu ku (2.0) vajaa vuoden.
		*so I’ve only smoked for (2.0) only under a year.*
29	EbE2:	°mm (.) mutta tota° ↑mut kyl se sit-
		°*mm (.) but erm*° ↑*but erm it-*
30		se jäi sitten myös niinku (.)
		*I was able to quit then (.)*
31		sen jälkeen kun päihteet jäi muutenkin
		*after I gave up all intoxicants*
32		pois ja näin ni sit se jäi(hhh)
		*and so that’s when I quit (hhh)*
33		kaikelle on tavallaan aikansa.
		*there’s a time for everything.*
34	EbE1:	ainaki itellä ollu hirveen tärkee huomata toi
		*at least for me it has been really important to notice that*
((10 lines removed: E1 describes how important it has been for her to notice that “there’s a time for everything” and some things can wait.))		
41		sit ne voi siellä odottaa sitä oikeeta-
		*then they can wait for the right.*
42	CLI1:	°oikeeta aikaa°.
		*° the right time°.*

When talking about the helpfulness of cigarette smoking, the client shows orientation toward recognizing its non-beneficial health effects. Thus, her orientation toward smoking could be characterized as ambiguous: cigarettes help release anxiety but are unhealthy. EbE1 picks up on this unhealthy side of smoking and orients toward the client’s need to quit smoking (line 5). Although the client does not topicalize her need to quit, EbE1 suggests that the client could “let the thought of quitting brew” (lines 8–9) even if the right time is not now. As in Extract 1, EbE1 presents herself as knowledgeable of how the rehabilitation process generally proceeds. She places the client’s situation on an imaginary timeline and shares knowledge about which things should be focused on in each phase of the process (lines 9–11).

At this point, EbE2 joins the discussion and shares her own experience, which is similar to that of the client. She describes her experience of the “worst times” as when she was in hospital (lines 12–16). Like the client, she needed the cigarettes to release anxiety and calm down her mood swings (lines 16–19). Even back at home, she needed to have a cigarette if anxiety woke her up at night (lines 20–22). Although EbE2′s experience is located in the past, it is constructed as a “second story” that recognizes the similarities between the experiences (Arminen, 2004). In her next turn, the client continues, still recognizing the similarity, with a minimal particle “joo” (translated as “yeah”) and a clitic particle *-kin* (*mullekin*, “I also,” line 23). The client changes the focus of her telling from the helpful effects of smoking to the problems it causes, but does not continue her experience-sharing. Instead, she defends herself by stating that she is not a “smoker”; she only started when she was hospitalized (lines 25–27), which was only under a year ago (line 28). In this way, the client is downgrading the relevance of smoking as a problem for herself.

EbE2 seems to orient toward the misalignment with the client’s turn. She starts with “but,” hesitates, and stammers (line 29) before revealing her solution: she was able to quit when she gave up all other intoxicants (lines 30–32). She concludes her point with an idiomatic expression “there’s a time for everything” (line 33). As Antaki (2007) have pointed out, the idiomatic expression allows the speaker to cast the private experience into the generally shareable public domain, and bring telling to a close. Topically, the idiomatic expression continues the line of thought that EbE1 initiated at the beginning of the extract: it would be good for the client not to worry about quitting smoking right now. EbE1 supports EbE2’s view. She states that it has been important for her to see this perspective and elaborates on the idea that some things can just be left to wait for “the right time.” In line 42, the client aligns with the EbE’s view by collaboratively completing her sentence (Lerner, 2004). In this way, the status of the sequence as a reciprocal experience-sharing sequence is emphasized.

To conclude, the EbEs were oriented toward mutual experience-sharing. They expressed their knowledgeability in relation to the client by, for instance, placing the client’s experience into a more general, temporarily longer frame, reformulating the client’s experience, and sharing their own solutions to the client’s problems. These asymmetrical actions were, however, immediately followed by the EbEs sharing experiences that were analogous to those of the clients, thus affiliating with them. In so doing, the EbEs were inviting the clients to talk more about their problems and to adopt more allowing, lenient perspectives toward their difficult situations. Typically, the clients aligned with these perspective shifts. However, as shown in Extract 2, misalignments were also possible. In such cases, the EbEs used idiomatic expressions to augment the general shareability of their experience-based perspective (see Antaki, 2007).

### Advising acceptance of illness: Compromising reciprocal experience-sharing

Although the EbEs generally oriented toward reciprocal experience-sharing, they sometimes advised the clients on how they should think and act in relation to their problem. After the clients’ descriptions of their problematic experiences, the EbEs reported their own current ways of thinking and acting. By not narrating their past experiences, and by showing their mutually shared experience with a client (see Extracts 1 and 2), the EbEs displayed less affiliation in relation to the client’s problem description (see Heritage, 2011 about less affiliative parallel and subjunctive assessments). By explicitly advising (see e.g. Vehvilainen, 2001) the clients on how they should think and act to be able to adopt more lenient thinking patterns about themselves, the EbEs also portrayed themselves as having specialized knowledge and superior epistemic authority (Heritage and Raymond, 2005).

Extract 3 shows an example of such cases. The client, who shares her experience, is a young adult. Before the extract takes place, she has told the EbE about her lack of interest in a driving license. Here, the client accounts for her lack of interest by referring to her age and a lack of motivation to drive (lines 1–4). Her dilemma is that she feels that a license is a must—something that every adult has and should have.

**Extract 3** (HW1, two EbEs, one client)

**Table:** 

01	CLI1:	mua ei kiinnostanu sillon ↑18-vuotiaana se (.)
		*I wasn’t interested when I was ↑18 (.)*
02		ja #vieläkää ei oo sillee# ollu tavallaan
		*and #still I haven’t # had in a way, any*
03		sellasta >motivaatioo tai sellasta mut sit
		>*motivation or anything but then on the other hand*
04		kuitenki aattelee et se ajokortti pitäis< olla,
		*I still think that a license is a must*<,
(42 lines removed: C tells the EBE that she needs the license for her future career plans and E2 tells how her assistant’s daughter also lacked motivation to get a driving a license)		
46	EbE1:	et sithän niitä pystyy
		*so then you can do it*
47		käymään niin ku tosi (.) tavallaan nopeesti,
		*like really (.) sort of quickly*,
48	CLI1:	↑tosi lohdullista kuulla et jotku muutki
		*really comforting to hear that others*
49		on sillee ollu niin ku <epämotivoituneita siihen>
		*have also been* <*unmotivated to get it it*>
50		tai silleen, koska niin kun mää oon vaan
		*or like, ‘cause I’ve just been, like*
51		potenu hirveetä syyllisyyttä ku kaikil kavereil
		*feeling so terribly guilty because all my friends*
52		oikeesti on se (.)>tai melkein kaikilla< (1.2)
		*have one (.)* >*or almost all* < *(1.2)*
53		niin sit sillee niin ku tuntenu ittensä jotenki
		*then I’ve like felt somehow*
54		↑huonommaks ihmisek(hh)s? ku ei oo ajokorttia.
		↑*a worse perso(hh)n? because I don’t have a license.*
55	EbE2:	[e:i e:i
		*[no: no*:
56	EbE1:	[ei se oo sitä] mää nykyää aina niin ku
		*[it’s not like that] nowadays I usually always*
57		yritän kysyä iteltäni että miks mun (.)
		*try to ask myself why should I (.)*
58		↑miks mun ois pakko, et jos on joku semmonen
		*↑why do I have to, like if there’s some*
59		asia mikä mua ei vaikka (.) >niin ku motivoi<?
		*thing that doesn’t (.)* >*like motivate me*<*?*
60		tietysti onhan elämässä jotain asioita. (.)
		*of course there are some things in life. (.)*
61		vessassa k(h)äyminen ja?
		*going to the toilet(h) and?*
62		hamp(hh)aitten pesu ja? s(h)yöminen ja?
		*brush(hh)ing your teeth and? eating(h) and?*
63	CLI1:	syö↑minen on ki[vaa,
		*ea↑ting is fu[n*,
64	EbE2:	[semmosia] (hhh) semmosia pakkoasioita
		*[kind of] (hhh) must-do-things*
65		mitä on niin ku ↑pakko tehä mut et
		*that ↑has to be done but that*
66		sitte aina miettis että
		*one should always think that*
67		no (.)↑miks mun on pakko ja sit joskus lähtee
		*well. (.)↑why do I have to and then sometimes you even start*
68		miettimään jo– jopa plussia ja miinuksia ja.
		*to think about the pros and cons and.*
69	EbE1:	nii ja onks se ainakaan niin ku tähän hetkeen se juttu.
		*yeah and at least if it’s necessary right now, the thing*
70	EbE2:	nii
		*yeah*
71	CLI1:	nii
		*yeah*

In a lengthy conversation that is not shown in the extract, the client explains the pressure she feels from school and friends to get a license. This makes EbE1 to tell a story about her friend’s daughter who also lacked motivation to get a license but managed to do it quickly when she really needed to. In line 46, she concludes the main point of her story: you can get a license when you need to and thus worrying about the lack of it is unnecessary.

The client responds by saying the story is “really comforting” (line 48). She has felt that almost all her friends have a license, and it has made her feel “terribly guilty” and a “worse person” (lines 49–54). Thus, in this case, the client discloses a problem and describes herself in negative terms. EbE2 responds to the client’s self-deprecation with denial (“no no no,” line 55) and EbE1 rejects the client’s interpretation as incorrect (“it’s not like that,” line 56). EbE1 continues by telling the client how she acts in similar situations: she always questions why she should have to do something that she finds unmotivating (lines 57–59). The beginning of EbE1’s telling is framed as a description of how she personally behaves (“I always try to ask myself,” line 57). She also highlights that this is something she would do “nowadays.” EbE1 provides a rather self-evident list of things one must do in life (lines 60–62), to which the client responds with humor “eating is fun” (line 63). After this, EbE2 advises the client on how she should think and act to be able to adopt a more accepting view of herself: “one should always think why do I have to” (lines 66–67). EbE2 uses an impersonal zero-person construction (translated as “one,” line 66) which leaves the actor unknown: it could be EbE2, the client, or anyone else. By leaving the reference open, EbE2 invites the client to identify with her recommendation (Leppanen, 1998), without defining the definite target of the piece of advice. It also invites the client to treat the advice as relevant and acknowledge it as a course of action to be followed (Vehvilainen, 2001). By giving advice, EbE2 positions herself epistemically differently from the prior two extracts. Here, EbE2 portrays herself as a person with specialized knowledge, adopting a superior epistemic status in relation to the client. EbE1 supports EbE2’s suggestion (line 69). The client provides a minimal response (line 71) and the sequence reached its closure.

In sum, rather than telling clients about their own experiences, the EbEs sometimes described their own current ways of thinking and acting. By explicitly advising the clients on how they should think and act to be able to adopt more lenient thinking-patterns toward themselves, the EbEs portrayed themselves as having specialized knowledge and superior epistemic authority in relation to the clients. After such advice, the clients stopped narrating their experiences and the sequence came to a close.

## Discussion

This article started with a description of the tension-laden positions of EbEs who enter environments of high professionality, such as specialized psychiatric health care. Through their own illness history and training and by accumulating experience from clients, EbEs become specialized—and increasingly professional—in their field (Jones, 2021). In interactive encounters with clients, the EbEs are instructed not to present themselves as experts (Hietala, 2016). The EbEs’ professionalism means balancing between the roles of experienced peer and expert in navigating the clients through the care system and through life (Davidson et al., 2012). We investigated the ways in an asymmetric, professional type of relationship with a reciprocal, mutuality-based equal relationship, is balanced through moment-to-moment interactions in a peer-based group intervention in psychiatric services.

We found that the EbEs reacted to the clients’ experience-sharing with two types of responses. In the first response type, they shared their own distressing experiences, on a long temporal continuum from the past to the present. The EbEs also revealed their own solutions to the mutually shared problems—either explicitly or implicitly, with reference to idiomatic expressions (see Antaki, 2007). In this way, they invited the clients to talk more about their problems and to adopt more lenient, accepting attitudes toward their difficult situations. The clients accepted the EbEs’ experience-sharing as affiliative (Extract 1), and this possibly helped them create a sense of hope (Davidson et al., 2012) and re-examine their expectations and plans for the future (Bury, 1982).

In the second response type, the EbEs described their present ways of thinking and acting and explicitly advised the clients how to think and act to be able to adopt more lenient thinking-patterns about themselves. After such advice, the clients stopped narrating their experiences and the sequence ended (Extract 3). Thus, it seems that by compromising the reciprocal experience-sharing, these responses were considered less affiliative in relation to the clients’ problem description (Heritage, 2011). By giving advice, the EbEs also moved toward an epistemically more asymmetric expert position (Vehvilainen, 2001). This is a position that the EbEs are instructed to refrain from (Hietala, 2016). As pointed out by Hietala (2016), “a peer should never advise or give guidance on how one should cope with their problems, but construes their own experiences and past reality, with both pain and humor” (pp. 388–389). It is important to note, however, that the responses, which involved “the mere sharing of an experience” (Extract 1 and 2), were also epistemically asymmetric. These experiences constituted a “past reality” for the EbE and a “current reality” for the client. This meant that the former observed the experience from a distance and the latter had no access to it. As this type of sharing of past and current experiences came across as highly affiliative, it suggests that at least some epistemic asymmetry may serve functional purposes and be essential in the interactions between EbEs and clients (see Pilnick and Dingwall, 2011).

Indeed, semi-professional, experience-based expertise seems to involve constant epistemic tensions as the participants struggle to keep the mutual orientation toward peer-based experience-sharing and affiliation. This became especially visible in the case of advice-giving (Extract 3), in which the EbEs displayed orientation toward its difficulty by evening out epistemic asymmetries. For instance, the EbEs delivered their advice without defining a clear target for it, including themselves as possible recipients (Leppanen, 1998). In this sense, the EbEs’ responses to the clients’ descriptions of their problems comes close to the professionalized practices in mental health care (Davidson et al., 2006), in which attempts to even out epistemic asymmetries are an essential aspect of client–professional encounters (Halonen, 2008; Weiste et al., 2016).

Our study had certain limitations. The small number of peer-to-peer group discussions (*n* = 3) in our data means that the interactional practices we found may well fail to represent all the ways in which EbEs respond to clients’ problem talk in psychiatric care, let alone in other social and health care contexts. However, in CA, the sufficiency of the sample size is not determined on the basis of the number of discussions (or participants) but on the adequacy of the data for the unique mode of investigation (O’Reilly and Parker, 2013). In our case, the data set was sufficiently comprehensive to enable us to identify recurring patterns in the EbEs’ responses to the clients’ problem talk (*n* = 29 cases). In this way, we were able to describe in detail some of the epistemic challenges that the EbEs may easily face when balancing expert and peer positions. As finding this balance may not be an easy task, providing a more nuanced understanding of real-life interactional patterns may help EbEs’ reflections on their evolving professional identities.

## Appendix

Transcription symbols (Jefferson, 2004)

[ ] Overlapping talk

(.) Pause: silence measured in seconds and tenths of a second

word Accented sound or syllable

- Abrupt cut-off of preceding sound

? Final rising intonation

, Final level intonation

. Final falling intonation

=Break and subsequent continuation of a single utterance

>text< Speech delivered more rapidly than usual

↑ Rising intonation

↓ Falling intonation

(h) Laughter in speech

#text# Creaky voice

## References

[bibr1-13634593231156822] Antaki C (2007) Mental-health practitioners’ use of idiomatic expressions in summarising clients’ accounts. Journal of Pragmatics 39(3): 527–541.

[bibr2-13634593231156822] Arminen I (2004) Second stories: The salience of interpersonal communication for mutual help in Alcoholics Anonymous. Journal of Pragmatics 36(2): 319–347.

[bibr3-13634593231156822] Bury M (1982) Chronic illness as biographical disruption. Sociology of Health & Illness 4(2): 167–182. 10260456 10.1111/1467-9566.ep11339939

[bibr4-13634593231156822] Clay S (2005) On Our Own, Together: Peer Programs for People With Mental Illness. Nashville, TN: Vanderbilt University Press.

[bibr5-13634593231156822] Davidson L Bellamy C Guy K , et al. (2012) Peer support among persons with severe mental illnesses: A review of evidence and experience. World Psychiatry 11(2): 123–128. 22654945 10.1016/j.wpsyc.2012.05.009 PMC3363389

[bibr6-13634593231156822] Davidson L Chinman M Sells D , et al. (2006) Peer support among adults with serious mental illness: A report from the field. Schizophrenia Bulletin 32(3): 443–450. 16461576 10.1093/schbul/sbj043 PMC2632237

[bibr7-13634593231156822] Drew P Chatwin J Collins S (2001) Conversation analysis: A method for research into interactions between patients and health-care professionals. Health Expectations 4(1): 58–70. 11286600 10.1046/j.1369-6513.2001.00125.x PMC5060048

[bibr8-13634593231156822] Enfield N (2011) Sources of asymmetry in human interaction: Enchrony, status, knowledge and agency. In: Stivers T Mondada L Steensig J (eds) The Morality of Knowledge in Conversation. Studies in Interactional Sociolinguistics. Cambridge: Cambridge University Press, pp.285–312.

[bibr9-13634593231156822] Halonen M (2008) Person Reference as a Device for Constructing Experiences as Typical in Group Therapy. In: Peräkylä A Antaki C Vehviläinen S , et al. (eds) Conversation Analysis and Psychotherapy. Cambridge: Cambridge University Press, pp.139–151.

[bibr10-13634593231156822] Heritage J (2011) Territories of knowledge, territories of experience: Empathic moments in interaction. In: Stivers T Mondada L Steensig J (eds) The Morality of Knowledge in Conversation. Studies in Interactional Sociolinguistics. Cambridge: Cambridge University Press, pp.159–183.

[bibr11-13634593231156822] Heritage J Raymond G (2005) The terms of agreement: Indexing epistemic authority and subordination in talk-in-interaction. Social Psychology Quarterly 68(1): 15–38.

[bibr12-13634593231156822] Hietala O (2016) Kokemusasiantuntija Kuntoutumisen Tukena [Expert-by-Experience in Support of Rehabilitation]. In: Autti-Rämö I Salminen AL Rajavaara M , et al. (eds) Kuntoutuminen [Rehabilitation]. Helsinki: Duodecim, pp.388–392.

[bibr13-13634593231156822] Jefferson G (2004) Glossary of transcript symbols with an introduction. In: Lerner G (ed.) Conversation Analysis: Studies From the First Generation. Amsterdam: John Benjamins, pp.14–31.

[bibr14-13634593231156822] Jones M (2021) Patient and public involvement in healthcare. Potentials and challenges of lay expertise and experiential knowledge. PhD Thesis, Tampere University, Finland.

[bibr15-13634593231156822] Jones M Pietilä I (2020) Personal perspectives on patient and public involvement – Stories about becoming and being an expert by experience. Sociology of Health & Illness 42(4): 809–824. 32072657 10.1111/1467-9566.13064

[bibr16-13634593231156822] Lee V (2018) The organisation of access in child mental health assessments: A conversation analysis of initial assessment appointments at a child and adolescent mental service. PhD Thesis, University of Leicester, UK.

[bibr17-13634593231156822] Leppanen V (1998) The straightforwardness of advice: Advice-giving in interactions between Swedish district nurses and patients. Research on Language and Social Interaction 31(2): 209–239.

[bibr18-13634593231156822] Lerner GH (2004) Collaborative turn sequences. Pragmatics and Beyond New Series 125: 225–256.

[bibr19-13634593231156822] Mead S Hilton D Curtis L (2001) Peer support: A theoretical perspective. Psychiatric Rehabilitation Journal 25(2): 134–141. 11769979 10.1037/h0095032

[bibr20-13634593231156822] Mikkonen I Saarinen A (2018) Vertaistuki Sosiaali- Ja Terveysalalla [Peer Support in Social and Health Care]. Helsinki: Tietosanoma.

[bibr21-13634593231156822] Munn-Giddings CD Borkman T (2017) Dialogic sharing of lived experience in different self-help/mutual aid groups. Groupwork 27(3): 26–46.

[bibr22-13634593231156822] O’Reilly M Parker N (2013) ‘Unsatisfactory saturation’: A critical exploration of the notion of saturated sample sizes in qualitative research. Qualitative Research 13(2): 190–197.

[bibr23-13634593231156822] Peng Z (2022) An epistemic illumination of the acceptance of advice in Chinese phone-in counseling for family problems. Discourse Studies 24(3): 291–306.

[bibr24-13634593231156822] Pilnick A Dingwall R (2011) On the remarkable persistence of asymmetry in doctor/patient interaction: A critical review. Social Science & Medicine 72(8): 1374–1382. 21454003 10.1016/j.socscimed.2011.02.033

[bibr25-13634593231156822] Schegloff EA (2007) Sequence Organization in Interaction. Cambridge: Cambridge University Press.

[bibr26-13634593231156822] Stevanovic M Frick M (2014) Singing in interaction. Social Semiotics 24(4): 495–513.

[bibr27-13634593231156822] Stevanovic M Svennevig J (2015) Introduction: Epistemics and deontics in conversational directives. Journal of Pragmatics 78: 1–6.

[bibr28-13634593231156822] Toikko T (2016) Becoming an expert by experience: An analysis of service users’ learning process. Social Work in Mental Health 14(3): 292–312.

[bibr29-13634593231156822] Vehvilainen S (2001) Evaluative advice in educational counseling: The use of disagreement in the “stepwise entry” to advice. Research on Language and Social Interaction 34(3): 371–398.

[bibr30-13634593231156822] Weiste E Leino T Laitinen J (2021) ‘No problem’ advice during occupational health examinations. Communication & Medicine 16(3): 280–291. 10.1558/cam.37849 39916034

[bibr31-13634593231156822] Weiste E Voutilainen L Peräkylä A (2016) Epistemic asymmetries in psychotherapy interaction: Therapists’ practices for displaying access to clients’ inner experiences. Sociology of Health & Illness 38(4): 645–661. 26574238 10.1111/1467-9566.12384

